# Effect of Tongqiao Huoxue Decoction Combined with Western Medicine on Ischemic Stroke: A Systematic Review

**DOI:** 10.1155/2020/8877998

**Published:** 2020-12-11

**Authors:** Da-yuan Zhong, Hao-yue Li, Lan Li, Ruo-meng Ma, Cheng-ting Jiang, Ding-xiang Li, Yi-hui Deng

**Affiliations:** ^1^Hunan University of Chinese Medicine, Changsha 410208, China; ^2^Hunan Provincial Brain Hospital, Changsha 410208, China

## Abstract

**Objective:**

We conducted a systematic review and meta-analysis to systematically evaluate the curative effect of Tongqiao Huoxue Decoction (TQHXD) combined with Western medicine treatment (WMT) on Ischemic Stroke (IS).

**Methods:**

Randomized controlled trials (RCTs) of TQHXD in the treatment of IS by computer retrieval of PubMed, Embase, Web of Science, Chinese Biomedical Literature Service System, CNKI, Wanfang Database, and Weipu Database. The retrieval time was taken from the establishment of the database to July 30, 2020. Two researchers, respectively, conducted a strict evaluation of the quality of the literature and extracted the data which were then entered in the RevMan5.3 software for meta-analysis.

**Results:**

40 articles were listed, which involved 3260 patients. Meta-analysis results show that TQHXD combined with WMT can significantly reduce patients' NIHSS score, serum hypersensitivity C-reactive protein (hs-CRP), plasma viscosity, serum fibrinogen, serum total cholesterol, and serum triglycerides and improve patients' ADL-Barthel scoring and treatment efficiency. However, there is no evidence that TQHXD combined with the WMT  group can significantly decrease the incidence of adverse events.

**Conclusion:**

The therapeutic effect of TQHXD combined with the WMT  group was significantly better than that of the WMT  alone group. For the treatment of patients with IS, TQHXD combined with WMT is worthy of application and promotion.

## 1. Introduction

Depending on the latest report from the World Health Organization, stroke is the second leading cause of death worldwide [1]. In 2016 alone, approximately 5.5 million people died of a stroke [2]. China is one of the countries with the heaviest stroke burden in the world. The latest census results showed that the incidence of stroke disease in China is about 1.6%, of which Ischemic Stroke (IS) accounts for 21.4% [3]. Evidence-based medicine research results [4] showed that most ischemic strokes are caused by blood clots blocking cerebral blood vessels. Elevated plasma fibrinogen is an important pathogenic factor of cerebrovascular diseases [5], and its increased content will cause plasma viscosity to rise, increase red blood cell and platelet aggregation, increase whole blood viscosity, and promote thrombosis, which leads to ischemia Sexual events. Bushi's studies have shown that thrombin activity is generally enhanced in patients with ischemic cerebrovascular disease and their high-risk groups [6]. Animal experiments [7] found that in the ischemic center of the rat model of middle cerebral artery occlusion, the activity of thrombin was considerably increased, and the expression of the prothrombin gene was upregulated. In vitro experiments [8] have shown that the nonproteolytic activity of thrombin could activate microglia and enhance microglia phagocytosis, thereby causing damage to neurons [9].

According to the symptoms and manifestations of IS, Chinese medicine believes that its onset is mainly caused by poor blood circulation [10, 11], so treatments that promote blood circulation are typically used for treatment. Tongqiao Huoxue Decoction (TQHXD) is derived from Wang Qingren's book *Medical Forest Correction*. Wang believed that it has the effects of promoting blood circulation, relieving pain, and refreshing. Studies [12] found that high-dose TQHXD could significantly improve the neuromotor function of rats with IS, increase serum NO and ET-1 levels, and reduce brain tissue Glu and Asp levels. TQHXD [13] may alleviate the neurological deficit in rats with cerebral ischemia and reperfusion by promoting the expression of neurotrophic factors in brain tissue and inhibiting lipid peroxidation. It can be seen that by looking for a suitable entry point, TQHXD combined with Western medicine treatment (WMT) can exert a beneficial effect. At present, a large number of clinical trials have confirmed the clinical efficacy of TQHXD in the treatment of IS. However, due to the different quality and sample size of the research, it lacks strong convincing power and lacks evaluation research on systematic safety and effectiveness. In order to provide an evidence-based reference for clinical medication, this article collects the published literature of TQHXD for the treatment of IS to conduct a systematic review.

## 2. Information and Methods

### 2.1. Research Registrations

We log in to PROSPERO official website (https://www.crd.york.ac.uk/PROSPERO/) to register a protocol for meta-analysis; the number of successful registration is CRD42020147688.

### 2.2. Types of Studies Included in the Study

Only clinical randomized controlled trials (RCTs) of TQHXD combined with WMT in the treatment of IS are included.

### 2.3. Research Objects Included in the Study

Patients with general clinical signs and symptoms of IS, excluding other diseases [14], are included. The course of the disease is within 2 weeks.

### 2.4. The Main Intervention Measures Are Included in the Study

The treatment group was TQHXD combined with the WMT  group, the control group was the WMT  group, and the two groups were both treated without acupuncture, cupping, massage, and other rehabilitation methods.

### 2.5. Outcome Indicators Are Included in the Study

We mainly extract the patient's NIHSS score, ADL-Barthel score, hsCRP (high-sensitivity C-reactive protein), plasma viscosity, fibrinogen, TC (total cholesterol), TG (triglycerides) before and after treatment, the treatment effective rate, and the incidence of adverse reactions. The calculation method [15] of the change of the mean value and the standard deviation is as follows, where *R* is a constant, and *R* is 0.5 in this study.

(1) $$\begin{array}{c} \text{mean}\left(\text{change}\right)=\text{mean}\left(\text{before}\right)-\text{mean}\left(\text{after}\right),\\ \text{sd}\left(\text{change}\right)=\sqrt{\text{sd}{\left(\text{before}\right)}^{2}+\text{sd}{\left(\text{after}\right)}^{2}-2^{*}R^{*}\text{sd}{\left(\text{before}\right)}^{*}\text{sd}\left(\text{after}\right)}. \end{array}$$

### 2.6. Research Exclusion Criteria

The exclusion criteria are as follows: the type of study does not correspond to nonrandomized controlled study, the intervention measures of the study do not match, the study is repeated, and the study does not contain 9 kinds of outcome indicators.

### 2.7. Retrieval Methods and Strategies

We search the following databases: PubMed database (https://pubmed.ncbi.nlm.nih.gov/), Embase database (https://www.embase.com), Web of Science database (http://webofscience.com), China Biomedical Document Service System (http://www.sinomed.ac.cn/), CNKI database (https://www.cnki.net/), Wanfang Database (http://www.wanfangdata.com.cn/index.html), and Weipu Database (http://www.cqvip.com/). Search terms: Stroke, Ischemic Stroke, Cerebral infarction, and Tongqiao Huoxue Decoction. Search time limit: database establishment to July 30, 2020. Search strategy: search terms are searched by free words and subject terms. Search strategy: search by free words and subject terms.

The search strategy in PubMed is given below.

   #1. Stroke [Title/Abstract]
   #2. Ischemic Stroke [Title/Abstract]
   #3. Cerebral Infarction [Title/Abstract]
   #4. Tongqiao Huoxue Decoction [Title/Abstract]
   #5. #1 or #2 or #3
   #6. #4 and #5

### 2.8. Data Extraction and Quality Evaluation

The documents retrieved from each database were entered into the CNKI-Estudy software to eliminate duplicates, read the title and abstract according to the PICOS principle, and then read the full text and finally determined to be included in the study. The quality evaluation relates to the Cochrane risk bias assessment tool. The obtained documents are provided and the quality was evaluated by two staff members (Lan Li and Ruo-meng Ma). Any differences during the period will be arbitrated by a third member (Da-yuan Zhong).

### 2.9. Statistical Analysis

RevMan5.3 software was used for data analysis. Binary variables use Odds Ratio (OR) and Relative Risk (RR); continuous variables use standard deviation (MD) as effective indicators. Using *t*^2^ and Chi^2^ statistics to analyze the heterogeneity between the studies, if *I*^2^ ≤50%, it indicates that the heterogeneity between the studies is small, and then the fixed-effects model is used. Otherwise, it indicates that the heterogeneity is large, and the random-effects model is used.

## 3. Results

### 3.1. The Search Results and the Basic Characteristics of the Included Studies

**Figure 1:**
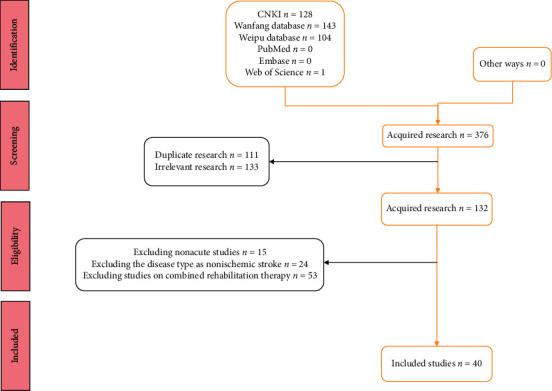
Flow chart of literature search inclusion.

A total of 40 RCTs meeting the criteria were included [16–55], involving 3260 patients. The literature search and inclusion flow chart is shown in Figure 1. The basic characteristics of the included studies are shown in Table 1.

### 3.2. Methodological Quality Evaluation

**Figure 2:**
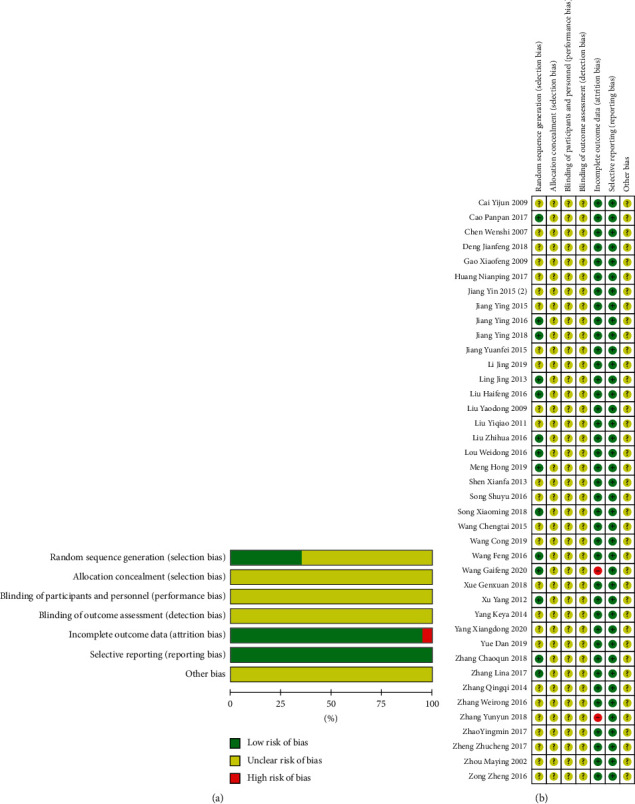
Literature quality evaluation of included studies.

14 studies [16, 17, 20, 21, 26, 29, 30, 33, 35, 38, 40, 43, 46, 48] mentioned specific random methods; all included studies [16–55]. No allocation concealment method or blinding method is mentioned, except for 2 studies [29, 36]. The rest of the studies have complete data and no selective reports; it is not clear whether there are other biases, as shown in Figures 2(a) and 2(b).

### 3.3. Meta-Analysis Results

#### 3.3.1. NIHSS Score

**Figure 3:**
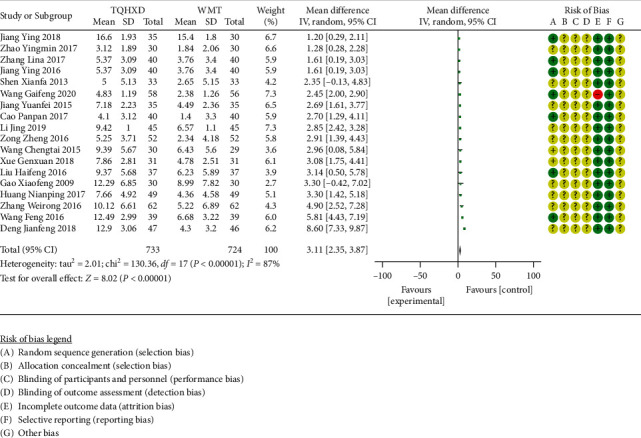
The forest diagram of TQHXD combined with WMT group and WMT group to compare the NIHSS score.

There are 18 studies [16, 17, 20, 25, 27, 29–31, 34, 37–41, 44, 45, 50, 51] that reported the NIHSS scoring index. The heterogeneity test results showed that there are significant differences in heterogeneity among the 18 studies, so the random-effects model was used for meta-analysis. The results (Figure 3) showed that the NIHSS score of the TQHXD combined with WMT group was significantly different from the group of WMT alone (MD = 3.11, 95% CI (2.35, 3.87), *P* < 0.00001).

#### 3.3.2. ADL-Barthel Score

**Figure 4:**
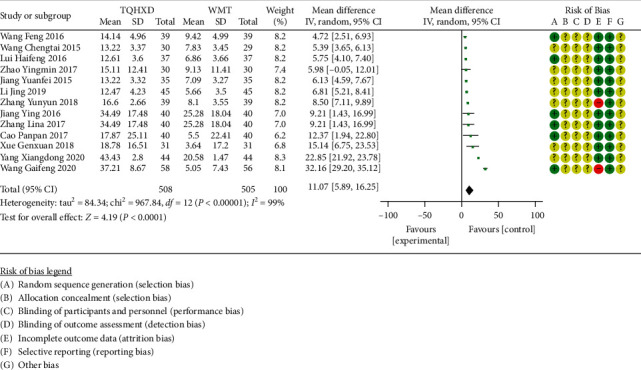
The forest diagram comparing TQHXD combined with WMT group and WMT group to compare the ADL-Barthel score.

There are 13 studies [17, 20, 27, 29, 30, 34, 38–40, 45, 47, 49, 50] that reported the ADL-Barthel score index, and the heterogeneity test results showed that there are significant differences in heterogeneity among these 13 studies. Therefore, the random-effects model was used for meta-analysis, and the results (Figure 4) showed that the TQHXD combined with the WMT group was significantly different from the group of WMT alone (MD = 11.07, 95% CI (5.89, 16.25), *P* < 0.00001).

#### 3.3.3. Hypersensitive C-Reactive Protein

**Figure 5:**
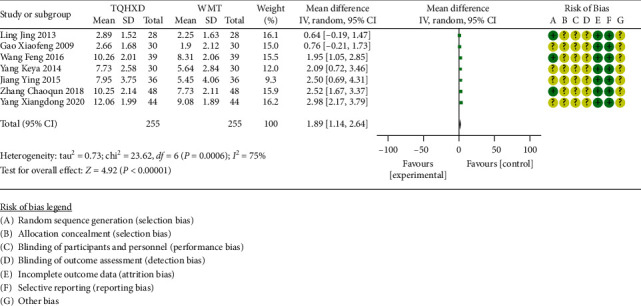
The forest diagram comparing TQHXD combined with WMT group and WMT group to compare the hsCRP.

There are 7 studies [18, 21, 28, 30, 31, 43, 47] that reported the index of high-sensitivity C-reactive protein. The heterogeneity test showed that there are significant differences in heterogeneity among the 7 studies, so the random-effects model was used for meta-analysis. The results (Figure 5) showed that the TQHXD combined with the WMT group was significantly different from the group of WMT alone (MD = 1.89, 95% CI (1.14, 2.64), *P* < 0.00001).

#### 3.3.4. Plasma Viscosity

**Figure 6:**
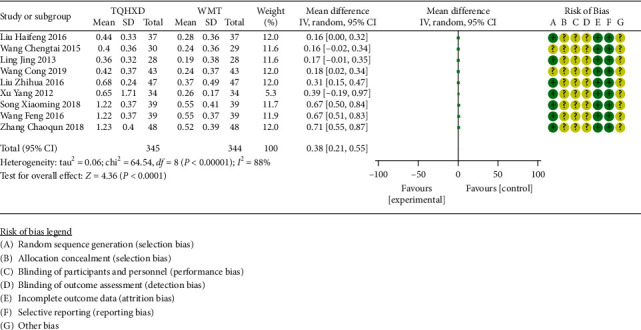
The forest diagram comparing TQHXD combined with WMT and WMT to compare fibrinogen.

There are 9 studies [21, 23, 26, 30, 33, 34, 38, 43, 46] that reported plasma viscosity indexes. The heterogeneity test results showed that there are significant differences in heterogeneity among these 9 studies, so the random-effects model was used for meta-analysis. The results (Figure 6) showed that the TQHXD combined with the WMT group was significantly different from the WMT group alone (MD = 0.38, 95% CI (0.21, 0.55), *P* < 0.00001).

#### 3.3.5. Fibrinogen

**Figure 7:**
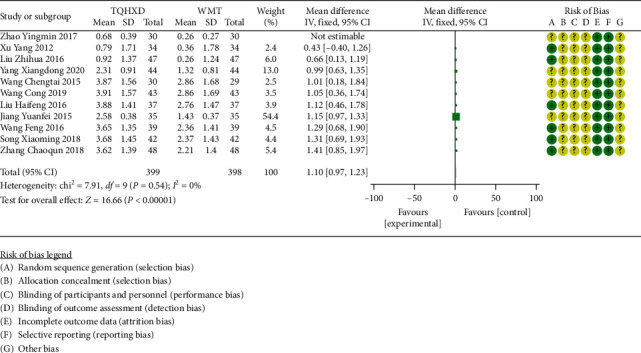
The forest diagram comparing TQHXD combined with WMT and WMT to compare the plasma viscosity.

There are 11 studies [21, 23, 26, 30, 33, 34, 38, 39, 45–47] that reported fibrinogen indicators, and the heterogeneity test results showed that there are none significant differences in heterogeneity among these 11 studies, so the fixed-effects model was used for meta-analysis. The results (Figure 7) showed that the TQHXD combined with the WMT group was significantly different from the WMT  group alone (MD = 1.10, 95% CI (0.97, 1.23), *P* < 0.00001).

#### 3.3.6. Serum Total Cholesterol

**Figure 8:**
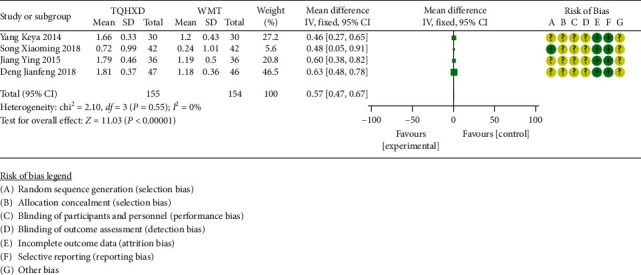
The forest diagram of TQHXD combined with WMT and WMT to compare TC.

There are 4 studies [19, 25, 26, 28] that reported serum total cholesterol indicators. The heterogeneity test results showed that there are none significant differences in heterogeneity among the four studies. Therefore, a fixed-effects model was used for meta-analysis. The results (Figure 8) showed that the TQHXD combined with the WMT group was significantly different from the WMT group alone (MD = 0.57, 95% CI (0.47, 0.67), *P* < 0.00001).

#### 3.3.7. Triglycerides

**Figure 9:**
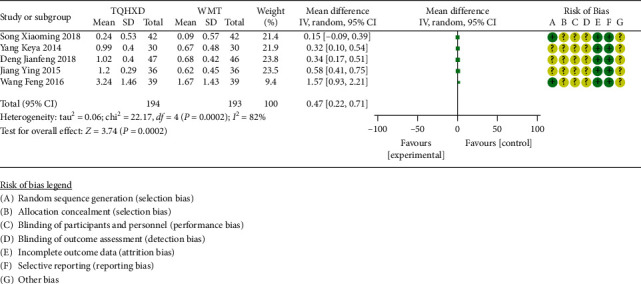
The forest diagram of TQHXD combined with WMT and WMT to compare TG.

There are 5 studies [19, 25, 26, 28, 30] that reported triglyceride indicators. The heterogeneity test results showed that there are significant differences in heterogeneity among the 5 studies. Therefore, a random-effects model was used for meta-analysis. The results (Figure 9) showed that the TQHXD combined with the WMT group was significantly different from the WMT  group alone (MD = 0.47, 95% CI (0.22, 0.71), *P* < 0.00002).

#### 3.3.8. Effectiveness of Treatment

**Figure 10:**
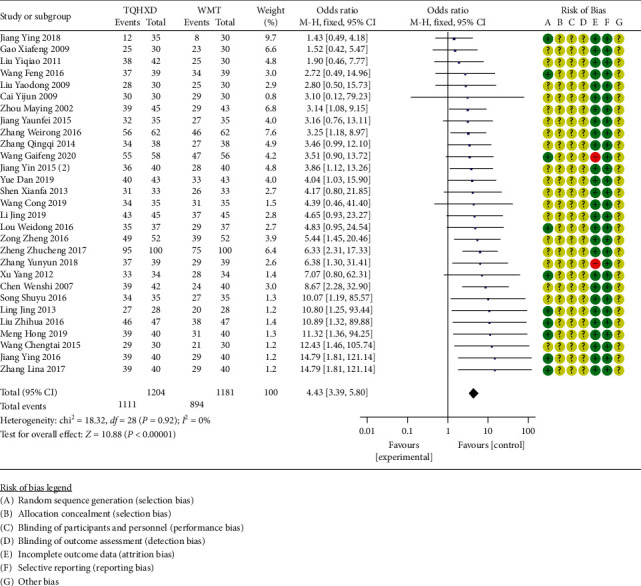
The forest diagram of TQHXD combined with WMT and WMT to compare the treatment efficiency.

There are 29 studies [16, 17, 19, 20, 22–24, 29–37, 42–46, 48–55] that reported effective rate indicators, and the heterogeneity test results showed that there are none significant differences in heterogeneity among these 29 studies, so a fixed-effects model was used for meta-analysis. The results (Figure 10) showed that the TQHXD combined with the WMT  group was significantly different from the WMT group alone (OR = 4.53, 95% CI (3.43, 5.99), *P* < 0.00001).

#### 3.3.9. Incidence of Adverse Reactions

**Figure 11:**
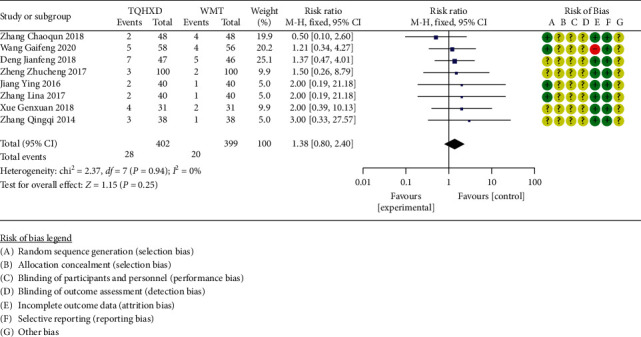
The forest diagram of TQHXD combined with WMT and WMT to compare the incidence of adverse reactions.

There are 10 studies [17, 20–22, 25–27, 29, 30, 42] that reported the incidence of adverse reactions. The heterogeneity test results showed that there are none significant differences in heterogeneity among these 10 studies, so the fixed-effects model was used for meta-analysis. The results (Figure 11) showed that the TQHXD combined with the WMT group and the WMT group were not significantly different from each other (RR = 1.38, 95% CI (0.80, 2.40), *P*=0.25).

### 3.4. Analysis of Publication Bias

**Figure 12:**
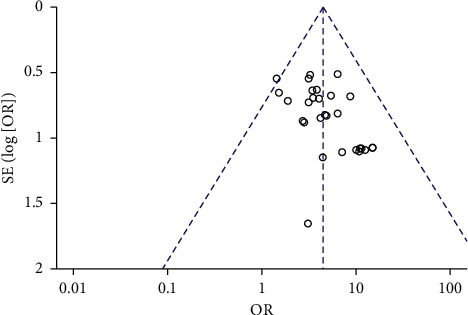
The funnel diagram of TQHXD combined with WMT and WMT to compare the treatment efficiency.

The effectiveness of 29 studies [16, 17, 19, 20, 22–24, 29–37, 42–46, 48–55] was evaluated by the funnel chart, and the results showed that the funnel chart has good symmetry, as shown in Figure 12, which means that there is no publication bias in the study.

## 4. Discussion

IS is a common cause of death and acquired disability [56]. Its pathogenesis is mainly due to slow blood flow, changes in blood composition, and increased blood viscosity on the basis of arteriosclerosis. The key factor for treatment is improving blood circulation in the ischemic area as soon as possible, eliminating secondary edema, and restoring the normal metabolism and nerve function of brain cells. The current treatment for this IS is mainly through intravenous drug thrombolysis, arterial thrombolysis, nonstent mechanical thrombectomy, and stent mechanical thrombectomy treatment [57]. Among them, intravenous drug thrombolytic treatment time is stricter (3–4.5 h in acute cerebrovascular occlusion), the recanalization rate of large artery occlusion is low, and the treatment effect is relatively poor. The cerebral infarction secondary to acute cerebrovascular occlusion is mostly caused by the formation of a large number of thrombi on the basis of cerebral artery stenosis. Even if the blood vessel is unblocked again after simple thrombolysis, the problem of obvious vascular stenosis will remain, and reocclusion is prone to occur [58]. Moreover, thrombolytic drugs could easily cause intracranial hemorrhage. The incidence of intracranial hemorrhage by intravenous thrombolysis is about 6.4% and that of arterial thrombolysis is close to 10%. This makes the condition worse, so the effect of treatment is not particularly ideal [59, 60]. Therefore, it is necessary to find more effective drugs to treat it. After thousands of years of development, Chinese medicine has left many classic prescriptions such as TQHXD and Buyang Huanwu Decoction, which have been used in the clinical treatment of human IS. Traditional Chinese medicine believes that the onset of IS is closely related to poor blood circulation and that blood stasis is both a pathological product and a pathogenic factor [11]. Modern physicians' understanding of the pathogenesis of IS by blood stasis and treatment methods for promoting blood circulation has become unified [61]. Poor blood circulation is considered to be the main cause of the disease, and the clinical application of the blood circulation method is effective. Significantly. TQHXD has the effects of promoting blood circulation, relieving pain, and resuscitating. It has been widely used in the treatment of brain injury diseases such as IS.

Meta-analysis is a statistical method that combines the effect size of the included studies of the same kind into a quantitative indicator. The combination can increase the sample size and improve the effectiveness of the test. Based on the meta-analysis method, this study combined 40 RCTs of TQHXD combined with WMT  in the treatment of IS. The results showed that TQHXD combined with WMT in the treatment of IS can significantly reduce the patient's NIHSS score, serum Sensitive C-reactive protein, plasma viscosity, serum fibrinogen, serum total cholesterol, and serum triglycerides which can improve the ADL-Barthel score of patients and the effective rate of treatment. But there is no evidence that TQHXD combined with WMT can significantly reduce the incident rate of adverse reactions. Li Lingfen's study [62] has shown that increased fibrinogen can enhance the aggregation of red blood cells and platelets and that increased levels of fibrinogen can cause increased plasma viscosity, thereby promoting thrombosis and leading to ischemic events. In addition, TC (total cholesterol) and LDL (low-density lipoprotein) are also related to the onset of cerebral infarction. Tao Fei's study [63] shows that reducing blood TC, TG, and LDL can prevent and reduce the occurrence of stroke. The existing research results show that TQHXD combined with WMT has an effect on fibrinogen, plasma viscosity, serum total cholesterol, triglycerides, and other indicators, thereby playing a therapeutic effect on IS. Du Yong's study [64] found that gavage of TQHXD can significantly increase the relative expression of LC3II/I, MnSOD, COXIV, and Bcl-2 protein in rats with brain injury and reduce the relative expression of p62 and cleaved caspase-3 protein. The amount indicates that the effect of TQHXD may be related to the activation of mitochondrial autophagy and inhibition of cell apoptosis. The results of adverse reaction analysis showed that there were 28 adverse events in the TQHXD combined with the WMT  group and 20 cases in the WMT group. The adverse reactions were mainly dizziness, fatigue, loss of appetite, nausea and vomiting, abdominal pain, and diarrhea. The adverse reaction symptoms were manifested between the two groups, and the difference was not statistically significant (*P* > 0.05). Therefore, it is not believed that the addition of TQHXD will increase the risk of adverse events. In summary, TQHXD combined with WMT has a good effect, which is worthy of application and promotion.

## 5. Limitation

However, there were some certain limitations. (1) The quality of the research methodology included in this systematic review is generally poor. The 40 included studies [16–55] did not mention whether blinding was implemented and it is not clear whether there are other biases. (2) The sample size of all studies is small, with only 3260 patients. (3) The adverse reaction reports are not detailed, and the specific symptoms and duration of adverse reactions are not described. (4) The number of studies included in this study is relatively small, where 14 studies [16, 17, 20, 21, 26, 29, 30, 33, 35, 38, 40, 43, 46, 48] mentioned specific random methods. (5) The number of included studies is small, all included studies are published with positive results, and it is impossible to determine whether there are unpublished negative results.

## 6. Conclusion

In summary, the safety and effectiveness of TQHXD combined with WMT in the treatment of IS still require a large number of RCTs with double-blind trials to verify. However, all studies of TQHXD combined with WMT in the treatment of IS were collected comprehensively, and the clinical efficacy of TQHXD combined with WMT in the treatment of IS was evaluated objectively. Therefore, the results of this study still have certain clinical guiding significance.

## Figures and Tables

**Table 1:** Basic characteristics of included studies.

Study	Therapy group				Control group				Course of treatment	Outcome indicators
	Treatment	Male/female	Age	Onset time	Treatment	Male/female	Age	Onset time		
JiangYing 2018 [16]	TQHXD + WMT	17/18	61.4 ± 6.7 y	—	WMT	17/13	60.6 ± 6.2 y	—	12 w	1 ⑧
JiangYing 2016 [17]	TQHXD + WMT	28/12	53.25 ± 6.35 y	8.51 ± 2.56 w	WMT	21/19	53.05 ± 6.01 y	8.27 ± 2.46 w	1 m	1 ②⑧⑨
Jiang Ying 2015 [18]	TQHXD + WMT	21/15	62.9 ± 12.4 y	12.6 ± 7.8 h	WMT	20/16	63.1 ± 12.1 y	13.2 ± 6.9 h	15 d	2 ⑥⑦
Jiang Ying 2015 [19]	TQHXD + WMT	22/18	58.6 ± 6.4 y	16.5 ± 5.6 h	WMT	21/19	60.3 ± 5.6 y	18.5 ± 6.4 h	—	⑧
Zhang Lina 2017 [20]	TQHXD + WMT	28/12	53.25 ± 6.35 y	8.51 ± 2.56 w	WMT	24/16	53.05 ± 6.01 y	8.27 ± 2.46 w	1 m	1 ②⑧⑨
Zhang Chaoqun 2018 [21]	TQHXD + WMT	29/19	62.85 ± 8.17 y	1.49 ± 0.37 d	WMT	26/22	62.90 ± 9.15 y	1.47 ± 0.78 d	2 w	2 ④⑤⑨
Zhang Qingqi 2014 [22]	TQHXD + WMT	20/18	59.3 ± 12.8 y	12.4 ± 3.6 h	WMT	22/16	60.4 ± 13.5 y	13.2 ± 4.7 h	4 w	⑧⑨
Wang Cong 2019 [23]	TQHXD + WMT	24/19	71.6 ± 2.9 y	37.6 ± 20.8 h	WMT	27/16	70.1 ± 2.6	36.8 ± 20.4 h	20 d	3 ⑤⑧
Cai Yijun 2009 [24]	TQHXD + WMT	20/10	50–80 y	<24 h	WMT	22/8	52–80 y	<24 h	30 d	⑧
Deng jianfeng 2018 [25]	TQHXD + WMT	27/20	61.80 ± 7.10 y	1.41 ± 0.38 d	WMT	26/20	60.90 ± 7.21 y	1.40 ± 0.30 d	1 m	1 ⑥⑦⑨
Song Xiaoming 2018 [26]	TQHXD + WMT	28/14	69.81 ± 5.78 y	9.36 ± 3.76 d	WMT	26/16	69.78 ± 5.76 y	9.32 ± 3.74 d	14 d	4 ⑨⑥⑦⑨
Xue Genxuan 2018 [27]	TQHXD + WMT	17/14	63.01 ± 15.33 y	5.01 ± 3.15 d	WMT	16/15	63.44 ± 15.21 y	5.75 ± 3.04 d	14 d	1 ②⑨
Yang Keya 2014 [28]	TQHXD + WMT	18/12	63.87 ± 6.87 y	—	WMT	20/10	64.43 ± 6.15 y	—	14 d	2 ⑥⑦
Wang Gaifeng 2020 [29]	TQHXD + WMT	29/31	44.6 ± 7.2 y	—	WMT	27/33	45.5 ± 7.6 y	—	14 d	1 ②⑧⑨
Wang Feng 2016 [30]	TQHXD + WMT	22/17	74.23 ± 8.56 y	8.87 ± 4.36 d	WMT	24/15	73.58 ± 9.42 y	8.25 ± 3.71 d	14 d	1 ②③④⑤⑦⑧⑨
Gao Xiaofeng 2009 [31]	TQHXD + WMT	18/12	57.4 ± 7.8 y	—	WMT	17/13	56.9 ± 8.7 y	—	14 d	1 ③⑧
Chen Wenshi 2007 [32]	TQHXD + WMT	24/18	47–82 y	—	WMT	20/20	46–81y	—	14 d	3 ⑤⑧
Liu Zhihua 2016 [33]	TQHXD + WMT	28/19	68.73 ± 3.97 y	4.91 ± 1.22 h	WMT	29/18	69.35 ± 4.12 y	4.37 ± 1.09 h	—	⑧
Wang Chengtai 2015 [34]	TQHXD + WMT	17/13	66.27 ± 5.53 y	4.02 ± 1.48 h	WMT	15/14	66.77 ± 4.84 y	3.80 ± 1.25 h	15 d	1 ②④⑤⑧
Lou Weidong 2016 [35]	TQHXD + WMT	20/17	64.3 ± 7.8 y	—	WMT	22/15	64.8 ± 7.6 y	—	28 d	⑧
Song Shuyu 2016 [36]	TQHXD + WMT	19/16	65.7士4.3 y	—	WMT	20/15	5.1士4.6 y	—	14 d	⑧
Zong Zheng 2016 [37]	TQHXD + WMT	33/19	66.4 ± 7.5 y	3.72 ± 0.63 d	WMT	31/21	65.8 ± 7.7 y	3.66 ± 0.74 d	14 d	1 ⑧
Liu Haifeng 2016 [38]	TQHXD + WMT	20/17	62.84 ± 5.15 y	13.61 ± 4.50 h	WMT	21/16	63.18 ± 5.62 y	13.27 ± 4.69 h	15 d	1 ②④
Zhao Yinmin 2017 [39]	TQHXD + WMT	13/17	61.52 ± 5.74 y	≤48 h	WMT	15/15	62.34 ± 6.15 y	≤48 h	14 d	①②⑤
Cao Panpan 2017 [40]	TQHXD + WMT	22/18	66.63 ± 10.01 y	—	WMT	20/20	67.30 ± 11.53 y	—	14 d	1 ②
Huang Nianping 2017 [41]	TQHXD + WMT	28/21	59.5 ± 6.4 y	≤72 h	WMT	30/19	58.9 ± 7.1 y	≤72 h	14 d	
Zheng Zhucheng 2017 [42]	TQHXD + WMT	53/47	65 ± 2.1 y	—	WMT	55/45	60 ± 2.5 y	—	14 d	⑧⑨
Ling Min 2013 [43]	TQHXD + WMT	-	46–80 y	4h-5 d	WMT	—	46–80 y	4 h-5 d	15 d	2 ④⑧
Shen Xianfa 2013 [44]	TQHXD + WMT	22/11	66.4 ± 5.7 y	<72 h	WMT	23/10	65.9 ± 6.3 y	<72 h	15 d	1 ⑧
Jiang Yuanfei 2015 [45]	TQHXD + WMT	18/17	74.83 ± 6.54 y	—	WMT	19/16	75.45 ± 5.37 y	—	14 d	1 ②④⑧
Xu Yang 2012 [46]	TQHXD + WMT	21/13	69.2 ± 7.1 y	4.2 ± 1.5 d	WMT	23/11	70.1 ± 7.3 y	4.3 ± 1.3 d	15 d	3 ⑤⑧
Yang Xiangdong 2020 [47]	TQHXD + WMT	28/16	67.22 ± 2.257 y	7.21 ± 0.45 h	WMT	28/16	67.21 ± 2.46 y	7.21 ± 0.47 h	14 d	2 ③
Meng Hong 2019 [48]	TQHXD + WMT	22/18	71.42 ± 3.86 y	8.63 ± 4.25 h	WMT	25/15	70.26 ± 3.76 y	8.12 ± 5.38 h	15 d	⑧
Zhang Yunyun 2018 [49]	TQHXD + WMT	29/10	58.6 ± 6.0 y	12.8 ± 1.8 h	WMT	16/13	59.5 ± 5.8 y	13.5 ± 2.0 d	7 d	3 ⑧
Li Jing 2019 [50]	TQHXD + WMT	26/19	73.8 ± 2.3 y	3.4 ± 0.7 d	WMT	24/21	74.4 ± 2.9 y	3.2 ± 0.4 d	30 d	1 ②⑧
Zhang Weirong 2016 [51]	TQHXD + WMT	32/30	61.4 ± 3.6 y	11.8 ± 2.1 h	WMT	34/28	62.1 ± 3.3 y	12.9 ± 2.5 h	15 d	1 ⑧
Yue Dan 2019 [52]	TQHXD + WMT	28/15	40–76 y	—	WMT	29/14	40–75 y	—	28 d	⑧
Liu Yiqiao 2011 [53]	TQHXD + WMT	20/22	56–85 y	<7 d	WMT	38/21	58–87 y	<7 d	20 d	⑧
Liu Yaodong 2009 [54]	TQHXD + WMT	19/11	38–79 y	2 h-3 d	WMT	22/8	40–76 y	2 h-3 d	—	⑧
Zhou Maying 2002 [55]	TQHXD + WMT	24/21	64.98 ± 11.61 y	10.51 ± 16.54 h	WMT	23/20	65.63 ± 10.55 y	10.84 ± 16.85 h	30 d	⑧

*Note.* TQHXD means Tongqiao Huoxue Decoction, WMT  means Western medicine treatment, *y* means year, *m* means month, *w* means week, *d* means day, and *h* means hour. In the outcome indicators, ① means NIHSS score, ② means ADL-Barthel score, ③ means hsCRP, ④ means plasma viscosity, ⑤ means fibrinogen, ⑥ means TC, ⑦ means TG, ⑧ means treatment effective rate, and ⑨ means adverse reaction rate.

## Data Availability

The data used in this article are obtained from public databases. The process including the literature, data extraction, and calculation are all described in the article. If necessary , the first author Da-yuan Zhong (13751728424@163.com) or the corresponding author Yi-hui Deng (644138330@qq.com) can be contacted to obtain data.
